# Biotechnological Fluorescent Ligands of the Bradykinin B_1_ Receptor: Protein Ligands for a Peptide Receptor

**DOI:** 10.1371/journal.pone.0148246

**Published:** 2016-02-04

**Authors:** Xavier Charest-Morin, François Marceau

**Affiliations:** Centre de recherche en rhumatologie et immunologie, CHU de Québec and Department of Microbiology-Infectious Disease and Immunology, Université Laval, Québec, QC, G1V 4G2, Canada; University of São Paulo, BRAZIL

## Abstract

The bradykinin (BK) B_1_ receptor (B_1_R) is a peculiar G protein coupled receptor that is strongly regulated to the point of being inducible in immunopathology. Limited clinical evidence suggests that its expression in peripheral blood mononuclear cells is a biomarker of active inflammatory states. In an effort to develop a novel imaging/diagnostic tool, we report the rational design and testing of a fusion protein that is a ligand of the human B_1_R but not likely to label peptidases. This ligand is composed of a fluorescent protein (FP) (enhanced green FP [EGFP] or mCherry) prolonged at its N-terminus by a spacer peptide and a classical peptide agonist or antagonist (des-Arg^9^-BK, [Leu^8^]des-Arg^9^-BK, respectively). The design of the spacer-ligand joint peptide was validated by a competition assay for [^3^H]Lys-des-Arg^9^-BK binding to the human B_1_R applied to 4 synthetic peptides of 18 or 19 residues. The labeling of B_1_R-expressing cells with EGFP or mCherry fused with 7 of such peptides was performed in parallel (microscopy). Both assays indicated that the best design was FP-(Asn-Gly)_n_-Lys-des-Arg^9^-BK; n = 15 was superior to n = 5, suggesting benefits from minimizing steric hindrance between the FP and the receptor. Cell labeling concerned mostly plasma membranes and was inhibited by a B_1_R antagonist. EGFP-(Asn-Gly)_15_-Lys-des-Arg^9^-BK competed for the binding of [^3^H]Lys-des-Arg^9^-BK to human recombinant B_1_R, being only 10-fold less potent than the unlabeled form of Lys-des-Arg^9^-BK to do so. The fusion protein did not label HEK 293a cells expressing recombinant human BK B_2_ receptors or angiotensin converting enzyme. This study identifies a modular C-terminal sequence that can be adapted to protein cargoes, conferring high affinity for the BK B_1_R, with possible applications in diagnostic cytofluorometry, histology and drug delivery (e.g., in oncology).

## Introduction

Bradykinin (BK)-related peptides, derived from the selective cleavage of circulating kininogens, stimulate two related G protein coupled receptors: the widely expressed BK B_2_ receptor (B_2_R) and the strongly regulated B_1_ receptor (B_1_R) [1]. These two entities can be distinguished using a number of properties. The B_2_R is responsive to the “native” kinins, BK and Lys-BK, produced by the kallikreins, whereas B_1_R has a selective affinity for a class of metabolites generated from native peptides by arginine carboxypeptidases, Lys-des-Arg^9^-BK (des-Arg^10^-kallidin) being the optimal agonist of the human B_1_R. Following stimulation, the B_2_R is phosphorylated and submitted to a cycle of β-arrestin-mediated endocytosis followed by extensive recycling, whereas the B_1_R is not phosphorylated and may be submitted to a modest lateral translocation to caveolae upon stimulation [2–4]. The B_2_R is constitutively expressed in vascular cells, non-vascular smooth muscle, sensory neurons and various epithelial cell types; the expression of the B_1_R is so strongly regulated that it appears to be inducible from a null or very low level of expression in most tissues. A model of historical importance to understand the inducible nature of B_1_R expression in inflammatory states is the intravenous injection of bacterial lipopolysaccharide in experimental animals: both the corresponding mRNA and vascular functional responses to exogenous B_1_R agonists are upregulated in a few hours following this treatment [5]. B_1_R expression is regulated in a rather complex matter via several signaling system that appear to induce transcription (NF-κB, Jak/Stat) and stabilization (protein kinase C) of the corresponding mRNA [6, 7]. B_1_Rs are therefore expressed during acute and chronic inflammation (including infection), ischemia, diabetes and neoplasia in response to cytokines and tissue injury in a wide range of models and contribute to various aspects of the physiopathology [1].

The present study addresses the following experimental question: in the absence of good anti-B_1_R antibodies that could detect the intact receptor at the cell surface, can a detection system based on a recombinant protein be developed? The proof-of-concept was based on fluorescent proteins (FPs) and a biotechnological/pharmacological approach based on the production and validation of selective ligands has been applied. Protein ligands may be intrinsically superior to peptides: peptides conjugated to cargoes such as chemical fluorophores or a radioisotope may bind to abundantly expressed peptidases, thus blurring the signal originating from the receptor. This has been illustrated by recently developed BK B_2_R ligands: carboxyfluorescein-aminocaproyl-BK is a low affinity agonist of the B_2_R that binds equally well to angiotensin converting enzyme (ACE) [8]. By contrast, enhanced green-fusion protein-maximakinin (EGFP-MK) is a much brighter, nanomolar affinity agonist of the rabbit B_2_R that does not bind to ACE [9]. An N-terminally extended peptide based on the B_1_R antagonist Lys-[Leu^8^]des-Arg^9^-BK that was conjugated to ^68^Ga apparently bound neutral endopeptidase in vivo [10]. A previously reported fluorescent B_1_R probe, B-10378 (carboxyfluorescein-aminocaproyl-Lys-des-Arg^9^-BK) [3] may also be prone to such non-specific interactions. We report the rational development of a high affinity fusion protein ligand of the BK B_1_R.

## Materials and Methods

### Vector design and construction

**Fig 1:**
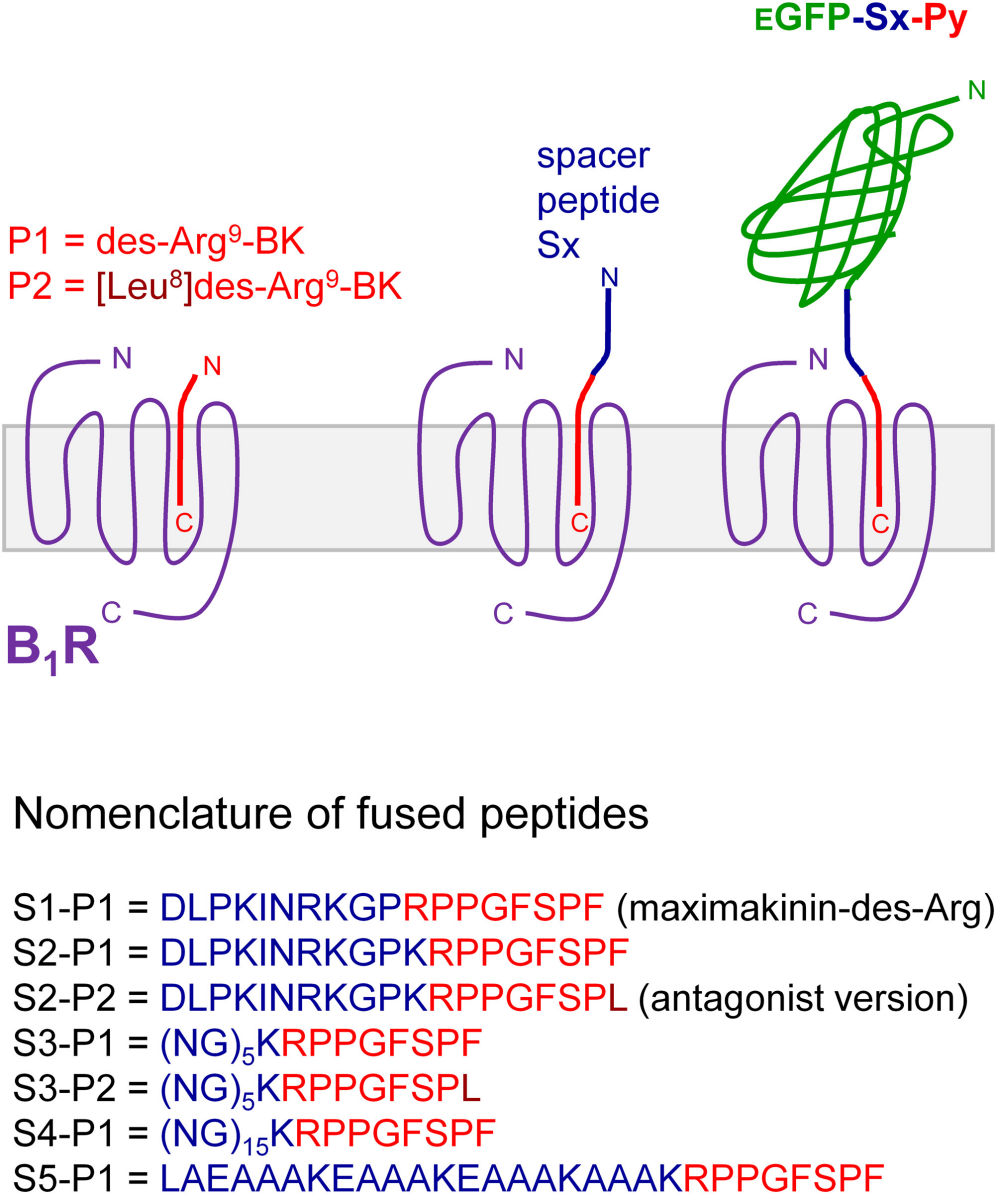
Schematic representation of the design of fusion proteins evaluated as potential B_1_R ligands. Two peptides sequences were fused to the C-terminus of enhanced green fluorescent protein (EGFP): one of 5 spacer peptides (designated S1 to S5) followed by a B_1_R ligand peptide (P1 agonist, P2 antagonist version).

The main starting material for B_1_R ligand construction was the pcDNA3.1 (-) vector containing the EGFP coding sequence; this vector determines the production of non-secreted cytosolic GFP and was C-terminally extended (Fig 1, general methods [9]). Alternatively, the same vector encoding mCherry was used in some experiments. The extension was composed of 2 modular sequences designated S (spacer) and P (ligand peptide). The latter sequences were either the minimal B_1_R agonist sequence (des-Arg^9^-BK = P1) or an antagonist version ([Leu^8^]des-Arg^9^-BK = P2) [1]. The first spacer peptide (S1) was derived from the amphibian peptide maximakinin that has been successfully used to design the B_2_R probe EGFP-MK [9]; thus, des-Arg^19^-maximakinin is S1-P1. The second (S2) design includes a lysine residue inserted in S1 at the N-terminal side of des-Arg^9^-BK or [Leu^8^]des-Arg^9^-BK, to take advantage of the higher affinity of Lys-des-Arg^9^-BK and derived peptides at the human form of the B_1_R [1]. The 3^rd^ linker design (S3 and S4) conserves this Lys residue, but the N-terminal extension is replaced by (Asn-Gly)_5 or 15_; the (Asn-Gly)_n_ linker has been successfully used as a spacer compatible with the extracellular fluid in recombinant protein construction [11]. Finally, the α-helix forming sequence, A(EAAAK)_3_A, previously documented to effectively separate domains of fusion proteins [12], was also tested as the spacer peptide S5 (Fig 1).

The vector EGFP-S1-P1 was produced by deleting the DNA sequence corresponding to the arginine codon at the C-terminal position of the EGFP-MK fusion protein coded by the previously reported EGFP-MK vector [9] using the technique described by Hansson et al. [13]. This deletion was realized using the following PCR primers: 5’-GGG TTC TCC CCT TTT TAA CTC GAG CAT GCA TCT-3’ (sense) and 5’-TG ATG CTC GAG TTA AAA AGG GGA GAA CCC CGG-3’ (antisense) followed by a DpnI digestion of the PCR product. The vector coding for the fusion protein EGFP-S1-P2 was generated by the substitution of the C-terminal phenylalanine found in the EGFP-S1-P1 fusion protein by a leucine. This was done by substituting the 3’ thymine (T) of the phenylalanine by an adenosine (A; TTT→TTA) using the following PCR primers: 5’-GT CCA CCG GGG TTC TCC CCT TTA TAA CTC GAG CAT GCA TCT AG-3’ (sense) and 5’-CT AGA TGC ATG CTC GAG TTA TAA AGG GGA GAA CCC CGG TGG AC-3’ (antisense) once again followed by a DpnI digestion.

Both vectors coding for EGFP-S2-P1 and EGFP-S2-P2 were produced by the insertion of a lysine residue, encoded by the AAG codon, at the C-terminal position of the S1 spacer, thus generating the S2 spacer. Using the oligonucleotides directed mutagenesis method, these vectors were generated using the following oligonucleotides: 5’-G ATC AAC CGC AAA GGA CCA AAG CGT CCA CCG GGG TTC TCC-3’ (sense) and 5’-GGA GAA CCC CGG TGG ACG CTT TGG TCC TTT GCG GTT GAT C-3’ (antisense). With these PCR primers, the EGFP-S2-P1 vector was generated from EGFP-S1-P1 while EGFP-S2-P2 was generated from EGFP-S1-P2.

To generate the EGFP-S3-P1 vector, the following oligonucleotides were annealed to obtain double strand DNA (dsDNA): 5’-G TAC AAG AAC GGC AAC GGC AAC GGC AAC GGC AAC GGC AAG CGT CCA CCG GGG TTC TCC CCT TTT TAA-3’ and 5’-G TAC TTA AAA AGG GGA GAA CCC CGG TGG ACG CTT GCC GTT GCC GTT GCC GTT GCC GTT GCC GTT CTT-3’. This dsDNA was then ligated in the BsrGI/XhoI digestion product of the EGFP-S1-P1 vector using the T4 ligase enzyme. The same strategy was used to produce the EGFP-S3-P2 vector but with the following oligonucleotides: 5’-G TAC AAG AAC GGC AAC GGC AAC GGC AAC GGC AAC GGC AAG CGT CCA CCG GGG TTC TCC CCT TTA TAA-3’ and 5’-G TAC TTA TAA AGG GGA GAA CCC CGG TGG ACG CTT GCC GTT GCC GTT GCC GTT GCC GTT GCC GTT CTT-3’.

The EGFP-S4-P1 vector was produced by the insertion of the following oligonucleotides in the spacer region of the EGFP-S3-P1. 5’-AAC GGC AAC GGC AAC GGC AAC GGC AAC GGC AAC GGC AAC GGC AAC GGC AAC GGC AAC GGC-3’. This insertion was realized by a PCR performed with the following primers: 5’-C GGC AAC GGC AAC GGC AAC GGC AAC GGC AAC GGC AAC GGC AAG CGT CCA CCG GG-3’ (sense) and 5’-CC CGG TGG ACG CTT GCC GTT GCC GTT GCC GTT GCC GTT GCC GTT GCC GTT GCC G-3’ (antisense). The goal of this insertion was to reduce the steric hindrance between the FP and the B_1_ receptor by prolonging the NG spacer. Due to the repetitive nature of this spacer, we obtained a fusion protein with 15 repeats (NG_15_) among other possible multiples of 5.

To generate the mCherry-S3-P1 vector, we used the Gibson assembly technique. The sequence coding for the mCherry protein was amplified from the Clontech vector using the following PCR primers: 5’-C GTT TAA ACG GGC CCT ATG GTG AGC AAG GGC GAG-3’ (sense) and 5’-TTG GTA CCG AGC TCG TTA AAA AGG GGA GAA CCC CGG TGG ACG CTT GCC GTT GCC GTT GCC GTT GCC GTT GCC GTT CTT GTA CAG CTC GTC CAT GC-3’ (antisense). The PCR product was then ligated in the XbaI/BamHI digestion product of the pcDNA3.1(-) vector using the Gibson Assembly Master Mix (New England Biology, Ipswich, MA) thus generating the mCherry-S3-P1 vector.

To produce the CherryFP-S5-P1 vector, the following oligonucleotide were annealed to generate dsDNA: 5’-G TAC AAG CTG GCG GAA GCG GCG GCG AAA GAA GCG GCG GCG AAA GAA GCG GCG GCG AAA GCG GCG GCG AAG CGT CCA CCG GGG TTC TCC CCT TTT TAA-3’ and 5’-A GCT TTA AAA AGG GGA GAA CCC CGG TGG ACG CTT CGC CGC CGC TTT CGC CGC CGC TTC TTT CGC CGC CGC TTC TTT CGC CGC CGC TTC CGC CAG CTT-3’. This dsDNA was then ligated in the BsrGI/HindIII digestion product of the mCherry vector using the T4 ligase enzyme. All the vectors coding for putative ligands of the B_1_R were validated by sequencing.

### Binding competition assay

[^3^H]Lys-des-Arg^9^-BK ([^3^H]des-Arg^10^-kallidin; 77.0 Ci/mmol) was purchased from PerkinElmer Biosciences, Boston, MA. The binding assays were performed using intact, adherent HEK 293a cells (24-well plates) as described [3]. The experiments dealt with the effect of cell coincubation of the radioligand at a fixed concentration (1 nM) with specific unlabeled peptides used in the design of fusion proteins (S1-P1, S2-P1, S3-P1 and S3-P2; Fig 1) and, in one case, with one of the fusion proteins. The 4 peptides were custom synthesized by Peptide 2.0 Inc. (Chantilly, VA) via standard solid-phase methodology and provided as >98.8% pure reagents (mass spectroscopy and HPLC analyses).

### Cell culture and production of biotechnological ligands

HEK 293a cells, obtained and grown as indicated [9], were used to produce the various non-secreted EGFP fusion proteins 48 h following transfection with the appropriate vector using a polyethyleneimine-based reagent as described [14]. The cytosolic proteins were produced as a lysate of HEK 293a as described [9]. Briefly, the producer cells were rinsed with phosphate buffered saline, left without supernatant, frozen for 2 hrs at -20°C, thawed and scraped. The resulting suspension was centrifuged (15,000 g, 10 min, 4°C) and the supernatant (the lysate) served as a concentrated stock of fusion proteins for biochemical and pharmacological characterization. A commercial GFP ELISA kit (Cell Biolabs, Inc., San Diego, CA) was applied as directed to quantify the concentration of GFP derivatives in lysates of various constructions (lysates diluted 1:500,000 to 1:2,500,000). A similar ELISA kit for mCherry (Cell Biolabs, Inc.) served to quantify fusion proteins containing this FP in lysates (dilution 1:100,000 to 1:1,000,000).

The institutional research ethics board (Comité d'éthique de la recherche, CHU de Québec—Université Laval, project 2012–323) approved the anonymous use of human umbilical cord segments obtained after normal deliveries. Mothers signed the corresponding informed consent form. Primary cultures of human umbilical artery smooth muscle cells (hUA-SMCs) were obtained and maintained precisely as described [6]. They expressed the marker α-actin (monoclonal antibody 1A4 from Sigma-Aldrich) and were used for experiments dealing with a naturally expressed and regulated population of B_1_Rs. 25-cm^2^ flasks of cells were treated for 16 hrs in their serum-containing medium with the combination of cytokines tumor necrosis factor-α + interferon-γ (R&D Systems, Minneapolis, MN and Cedarlane Labs. Ltd., Hornby, ON, Canada, respectively) to upregulate the B_1_R population [7]. Then, cells were stimulated for a 20-min incubation period (37°C) with B_1_R ligands to assay a documented acute response of vascular smooth muscle to agonists of this receptor: the acute dephosphorylation of AKT (protein kinase B) [15]. After extraction, immunoblots based on polyclonal anti-phospho-Ser^473^-AKT and polyclonal anti-AKT antibodies (Cell Signaling Technologies) were performed as described [15].

### Generation of the B_2_R-mCherry vector

First, the sequence corresponding to the human B_2_R was amplified from the previously described human B_2_R vector [16] using the following PCR primers: 5’-C GTT TAA ACG GGC CCT ATG CTC AAT GTC ACC TTG CAA G-3’ (sense) and 5’-CTC ACC ATC TGT CTG CTC CCT GCC CCA-3’ (antisense). Then, the sequence coding for the mCherry was in turn amplified with the following primers: 5’-GC AGA CAG ATG GTG AGC AAG GGC GAG-3’ (sense) and 5’-TTG GTA CCG AGC TCG TTA TCT TAG ATC CGG TGG ATC CC-3’ (antisense). Using the Gibson assembly technique, both fragments were ligated in the XbaI/BamHI digestion product of the pcDNA3.1(-) vector to generate the B_2_R-mCherry vector. This vector was validated by sequencing.

### Generation of the ACE-mCherry vector

The sequence encoding for the ACE was amplified by PCR from a vector coding for the human form of this enzyme (gift by Dr. P. Corvol, Paris, France) with the following primers: 5’-C GTT TAA ACG GGC CCT ATG GGG GCC GCC TCG GGC-3’ (sense) and 5’-CT CAC CAT GGA GTG TCT CAG CTC CAC CTC GGA GC-3’ (antisense). Then, the sequence coding for the mCherry FP was in turn amplified with the following primers: 5’-GA CAC TCC ATG GTG AGC CAA GGG CGA G-3’ (sens) et 5’-TTG GTA CCG AGC TCG TTA TCT AGA TCC GGT GGA TCC C-3’ (anti-sens). Using the Gibson assembly technique, both fragments were ligated in the XbaI/BamHI digestion product of the pcDNA3.1(-) vector to generate the ACE-mCherry vector. This vector was validated by sequencing.

### Cell stimulation and analysis

Other recipient HEK 293a cells were grown and transiently transfected as described above with a vector coding for the human B_1_R C-terminally tagged with FLAG (hB_1_R-FLAG) [14], a pharmacologically intact B_1_R construction, or with human B_2_R or ACE fused at their C-terminus to the mCherry FP. Stimulations for microscopic or cytofluorometric experiments were based on the lysates for the GFP/mCherry-based ligand constructs. Receptor- or ACE- expressing HEK 293a cells were generally treated for 30 min with stimulants (incubation carried out at 37°C in humidified atmosphere containing 5% CO_2_), rinsed 3 times with phosphate buffered saline, observed in microscopy for epifluorescence and photographed using an Olympus BX51 microscope coupled to a CoolSnap HQ digital camera (filters for GFP: excitation 460–500 nm, emission 510–560 nm; for mCherry FP: excitation 525–555 nm, emission 600–660). The objective lens was the 100× oil UPlanApo (Olympus).

### Immunoblots

The identity and concentration of cytosolic GFP proteins was verified in immunoblotting experiments (1–10 μl of cell lysate loaded per track; 9–15% SDS-polyacrylamide gels) using the monoclonal anti-GFP antibodies JL8 (Clontech, Palo Alto, CA) coupled with an appropriate HRP-conjugated secondary antibody [9]. The monoclonal anti-mCherry FP antibody directly conjugated to HRP, clone 1C51 (Abcam) served the same purpose for fusion proteins containing this FP.

### Drugs

Unlabeled Lys-des-Arg^9^-BK was purchased from Sigma-Aldrich (St. Louis, MO). Compound 11 (2-{(2R)-1-[(3,4-dichlorophenyl)sulfonyl]-3-oxo-1,2,3,4-tetrahydroquinoxalin-2-yl}-N-{2-[4-(4,5-dihydro-1H-imidazol-2-yl)phenyl]ethyl}acetamide) is a powerful antagonist at the human and rabbit B_1_ receptor [17] (gift from Dr. D. J. Pettibone, Merck Research Laboratories, West Point, PA).

### Data analysis

Numerical values are means ± S.E.M. In the photographic record of microscopic studies, the cellular fluorescence was quantified by manually delineating each cell (minimally identified by its autofluorescence) and recording the mean pixel intensity (0–255 scale) of the selections using the Photoshop software (version 6, Adobe, San Jose, CA). These numerical values were averaged and compared using ANOVA followed by Dunnett’s test to compare experimental groups with a common control value or with Tukey-Kramer multiple comparison test to compare all pairs of values (InStat 3.05, GraphPad Software Inc., San Diego, CA). Data from the radioligand competition assays were fitted by nonlinear regression to a one-site competition equation to derive IC_50_ values and their 95% confidence limits (C.L.) (Prism 4.0, GraphPad Software Inc.).

## Results

### B_1_R affinity of the candidate peptides for fusion protein design

**Fig 2:**
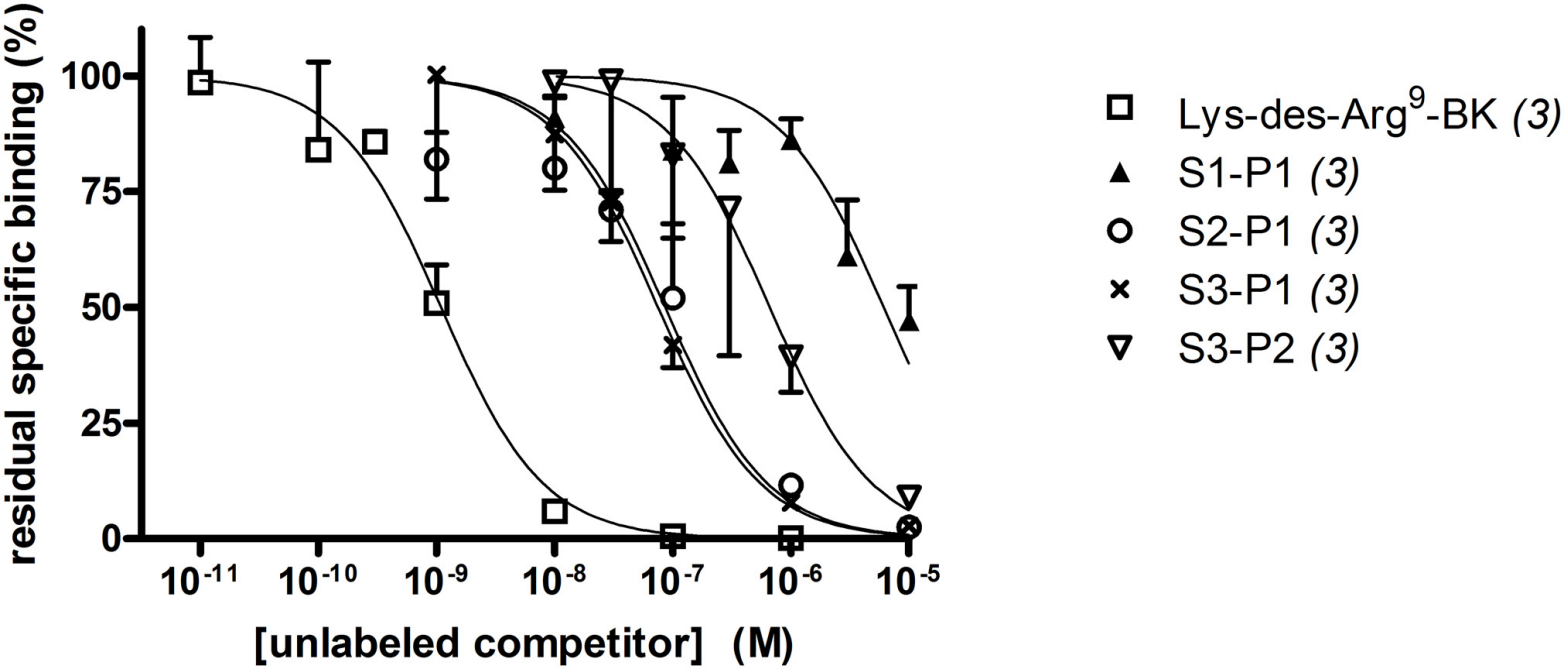
Competition of [^3^H] Lys-des-Arg^9^-BK (1 nM) binding to hB_1_R-FLAG by a panel of unlabeled peptides. (verification of the affinity of selected peptides used in fusion protein design for the B_1_R ligands). Specific binding values (percent of control) are the means ± S.E.M. of the number of duplicate determinations indicated between parentheses. Derived numerical affinity estimates are reported in Results.

While unlabeled Lys-des-Arg^9^-BK very efficiently competed for the binding of its own tritiated form to hB_1_R-FLAG (IC_50_ = 1.09 nM, 95% C.L. 0.66–1.79 nM), 4 N-terminally prolonged sequences exhibited widely variable affinities (Fig 2). In the agonist series, synthetic S1-P1 (the des-Arg form of maximakinin, Fig 1) was a weak competitor (IC_50_ = 6.11 μM, 95% C.L. 3.5–10.7 μM), but the insertion of the Lys residue in S2-P1 increased the affinity (IC_50_ = 86.3 nM, 95% C.L. 52.5–142 nM). The peptide that conserved the Lys-des-Arg^9^-BK sequence N-terminally extended with the Asn-Gly repeat, S3-P1, had a slightly higher affinity (IC_50_ = 75.1 nM, 95% C.L. 51.2–110 nM). The antagonist version of the latter peptide, S3-P2, lost much affinity (IC_50_ = 663 nM, 95% C.L. 409–1076 nM) vs. the agonist S3-P1. The 8.6-fold loss of affinity for S3-P2 vs. S3-P1 is reminiscent of the 4.8-fold inferior absolute affinity of Lys-[Leu^8^]des-Arg^9^-BK vs. Lys-des-Arg^9^-BK for the human recombinant B_1_R established using a similar radioligand binding competition assay [18].

### Expression of fusion EGFP-peptide proteins

**Fig 3:**
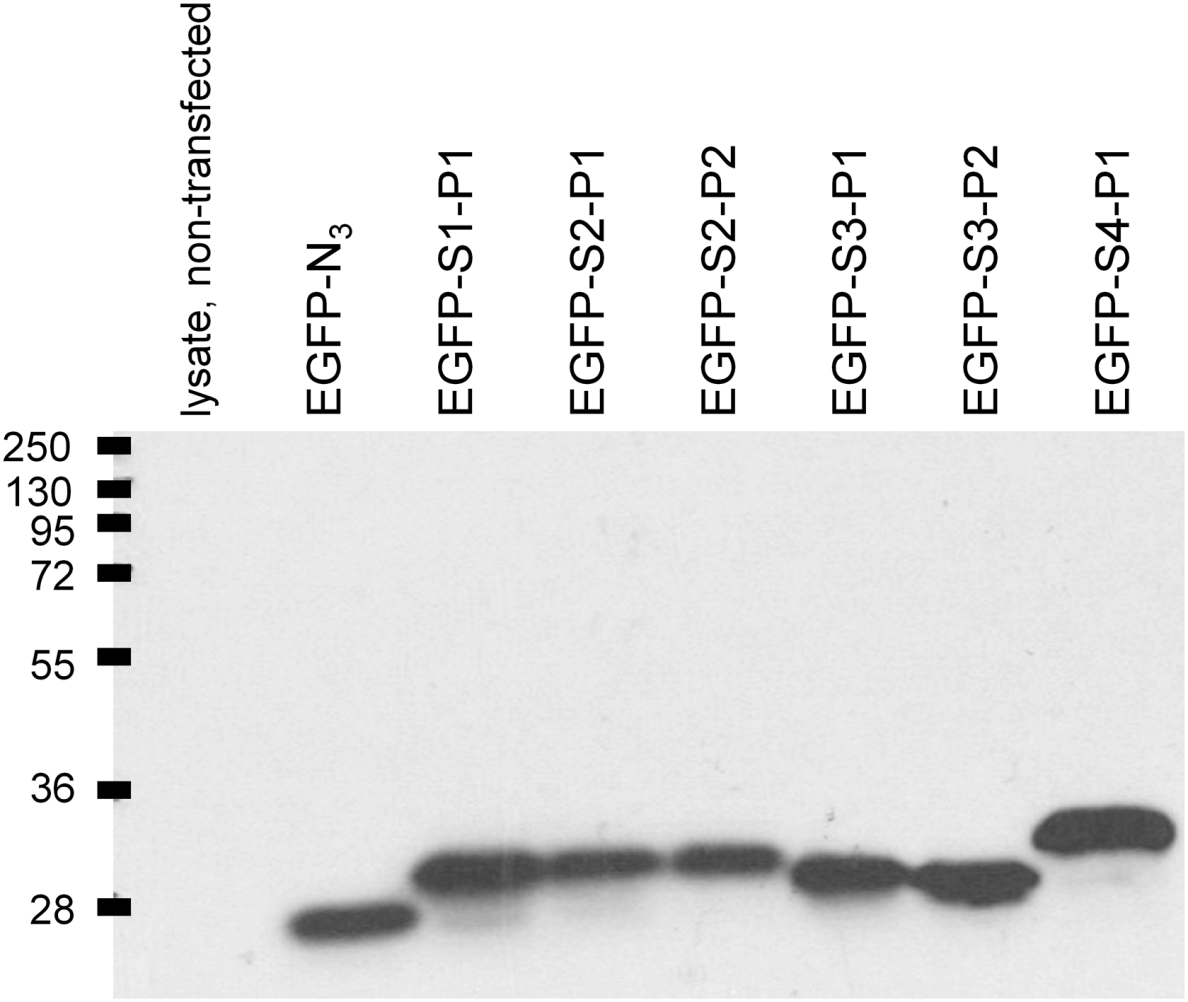
Immunoblots of the cell lysates of HEK 293a cells transiently expressing either EGFP-N_3_ (authentic GFP, 27 kDa, 10 μl lysate) or fusion proteins based on EGFP (1 μl lysate per track migrated in a 15% gel). For each of the fusion protein, the major band is the heaviest and is consistent with the predicted size of the insert (S1-P1: 2.02 kDa; S2-P1: 2.15; S2-P2: 2.12; S3-P1: 1.89; S3-P2: 1.85; S4-P1: 3.79). Representative blot from 3 separate experiments.

Producer HEK 293a cells expressing the EGFP proteins extended with specific sequences were highly fluorescent as these proteins are distributed in all the cellular water (data not shown; as in [9] for EGFP-MK). The lysates of HEK 293a cells that transiently expressed EGFP-Sx-Py constructions contained micromolar concentrations of the fusion proteins based on an ELISA for GFP (3.2–16 μM, the longer construction EGFP-S4-P1 usually being at the lower end of the range). The homogeneity and molecular masses of the constructions has been estimated by immunoblotting cell lysates with the anti-GFP monoclonal antibody JL8 (Fig 3). Consistent with the high stability of GFP in mammalian cells [19], cells transfected with the Clontech vector corresponding to authentic EGFP exhibited a single band at 27 kDa. The major heavier bands present in the lysates of cells expressing the EGFP fusion proteins exhibited molecular weight shifts consistent with the predicted additional masses determined by the fused peptide sequences: 2.02 kDa (S1-P1), 2.15 (S2-P1), 2.12 (S2-P2), 1.89 (S3-P1), 1.85 (S3-P2) and 3.79 (S4-P1) (Fig 3). S4-P1 possessed an extended (NG)_15_ repeat and was produced to test the effect of the spacer length.

### Screening of potential biotechnological ligands of the B_1_R in a microscopic assay

**Fig 4:**
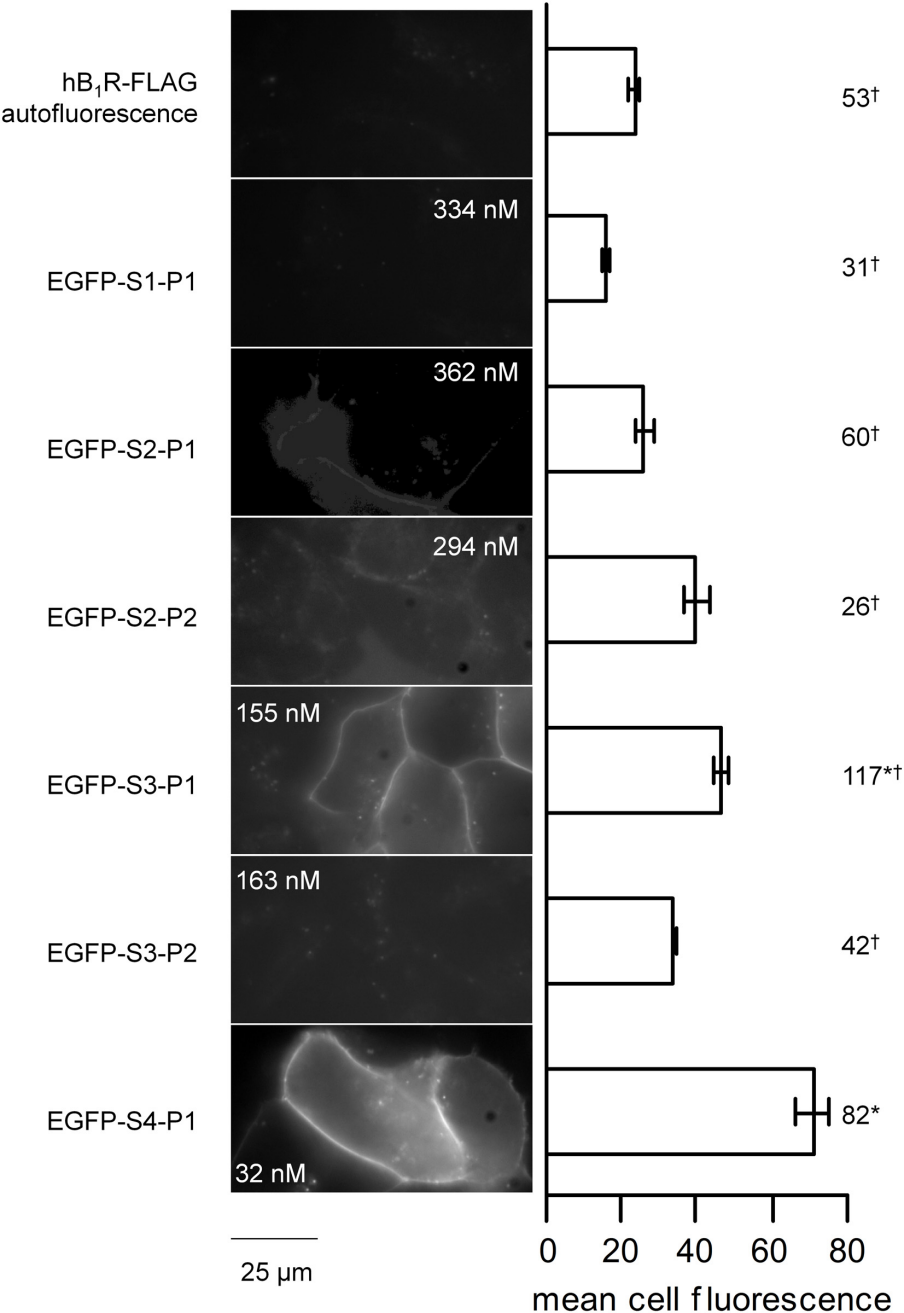
Tests for microscopic labeling of HEK 293a that transiently express the hB_1_R-FLAG construction using lysates of other producer cells that expressed EGFP fusion proteins. (nomenclature as in Fig 1). Left: Cells were treated for 30 min at 37°C with fusion proteins in the form of diluted (1:100–1:40) lysates of other producer cells. The estimated final concentration of the fusion protein is indicated in microscopic fields (from the lysate concentrations estimated using a GFP ELISA). Representative results of multiple fields. Green epifluorescence (1000 ×, 4-sec exposition). Right: histograms represent the averaged cellular fluorescence intensities in the photographic record (estimated as described in Materials and methods). The number of evaluated cells is indicated at the right hand side. ANOVA indicated that the values were heterogeneous (P<10^−4^). Comparison of values with the common control value (autofluorescence, top-most histogram) * P<0.001; comparison with the highest signal, EGFP-S4-P1: † * P<0.001 (Bonferroni multiple comparison test).

The lysate of producer cells was applied to other HEK 293a recipient cells transiently expressing hB_1_R-FLAG (Fig 4). The des-Arg form of EGFP-MK (= EGFP-S1-P1) failed to label the intact cells, while the insertion of the Lys that confers high affinity for the ligand Lys-des-Arg^9^-BK in this sequence (EGFP-S1-P1) cause discernible but weak plasma membrane labeling (Fig 4). Replacing the spacer peptide of amphibian origin by (Asn-Gly)_5_ while retaining the Lys residue was more successful, with a bright labeling of many cells that expressed hB_1_R-FLAG. The subcellular distribution of the fluorescence predominantly concerned the plasma membrane, in sharp contrast with the endosomal location of EGFP-MK in cells that express rabbit B_2_Rs [9]. [Leu^8^]des-Arg^9^-BK is a competitive antagonist of kinins at the B_1_R and its potency is increased at the human B_1_R by including the Lys residue at the N-terminus of the sequence [1]. Human B_1_R-FLAG-expressing cells treated with the putative antagonist version of two construction (S2 or S3 coupled to P2 = [Leu^8^]des-Arg^9^-BK) were not labeled over the cell autofluorescence level (Fig 4), consistent with a critical loss of receptor affinity in synthetic S3-P2 vs. S3-P1 (Fig 2). EGFP-S4-P1 (EGFP-(NG)_15_-Lys-des-Arg^9^-BK, final concentration 32–74 nM) produced the brightest labeling of B_1_R-FLAG-expressing cells (Fig 4).

**Fig 5:**
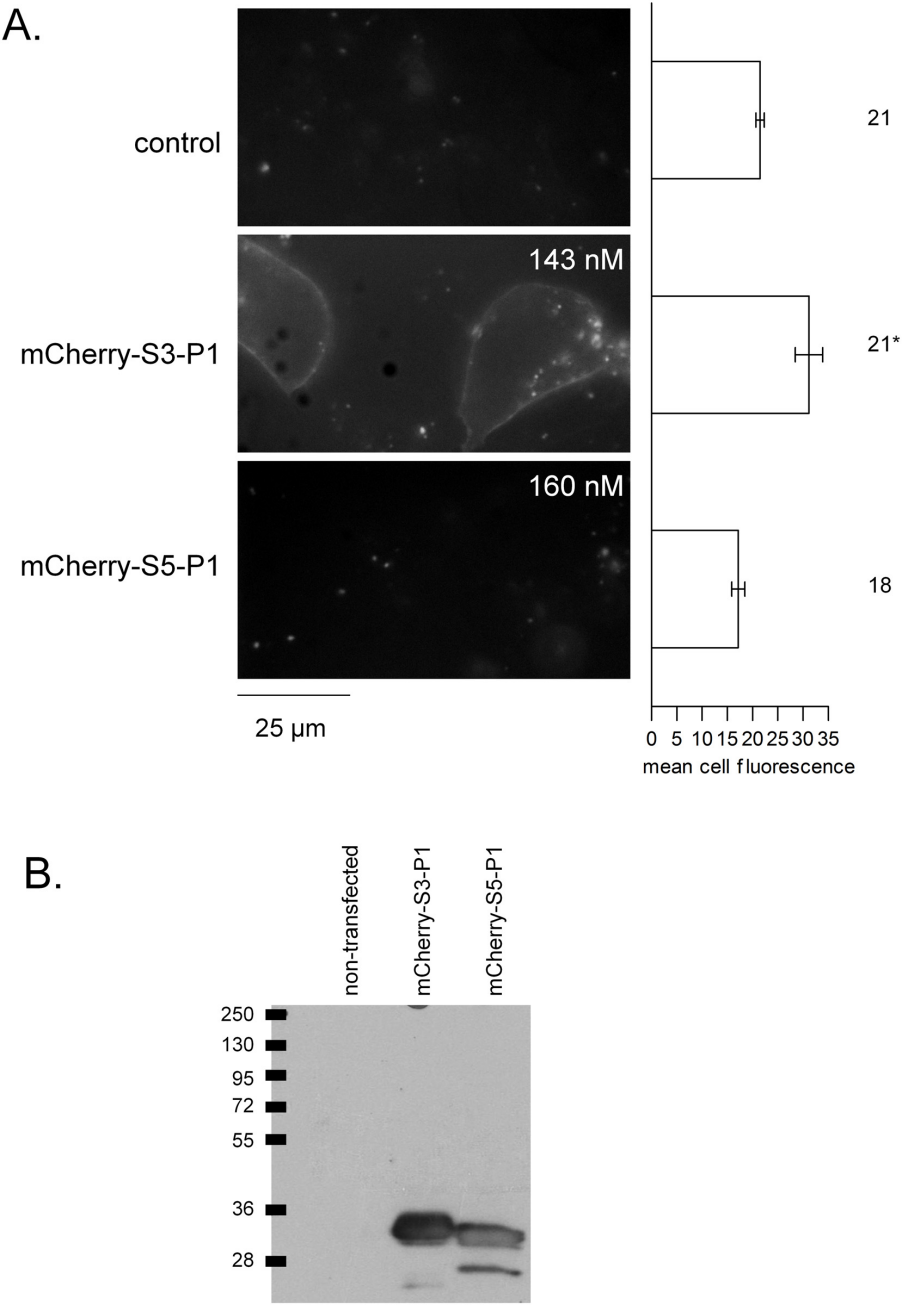
mCherry fusion proteins as B_1_R ligands. A. Tests for microscopic labeling of HEK 293a that transiently express the hB_1_R-FLAG construction using lysates of other producer cells that expressed mCherry fusion proteins (nomenclature as in Fig 1, presentation as in Fig 4, microscopy results duplicated in separate batches of cells). ANOVA indicated that the mean cellular fluorescence intensities were heterogeneous (P<10^−4^). Comparison of values with the common control value (autofluorescence, top-most histogram) * P<0.01 (Dunnett’s test). B. Immunoblots of the cell lysates of HEK 293a cells transiently expressing either mCherry fusion protein using an anti-mCherry monoclonal antibody. Presentation as in Fig 3.

In order to document the flexibility of the fluorescent peptide design, one efficacious spacer-peptide (S3-P1) was fused to the alternate red FP mCherry (Fig 5A). This construct also labelled HEK 293a cells that expressed the recombinant human B_1_R-FLAG, mainly at the level of the plasma membrane. By comparison, the α-helix forming spacer peptide was tested under the form of mCherry-S5-P1: the corresponding lysate did not label plasma membrane B_1_Rs (Fig 5A). Both proteins were expressed at similar levels (ELISA for mCherry, values of final dilutions reported in Fig 5) and were represented by one ~30 kDa major band in immunoblots exploiting anti-mCherry monoclonal antibodies (Fig 5B).

### Further study of EGFP-S4-P1

**Fig 6:**
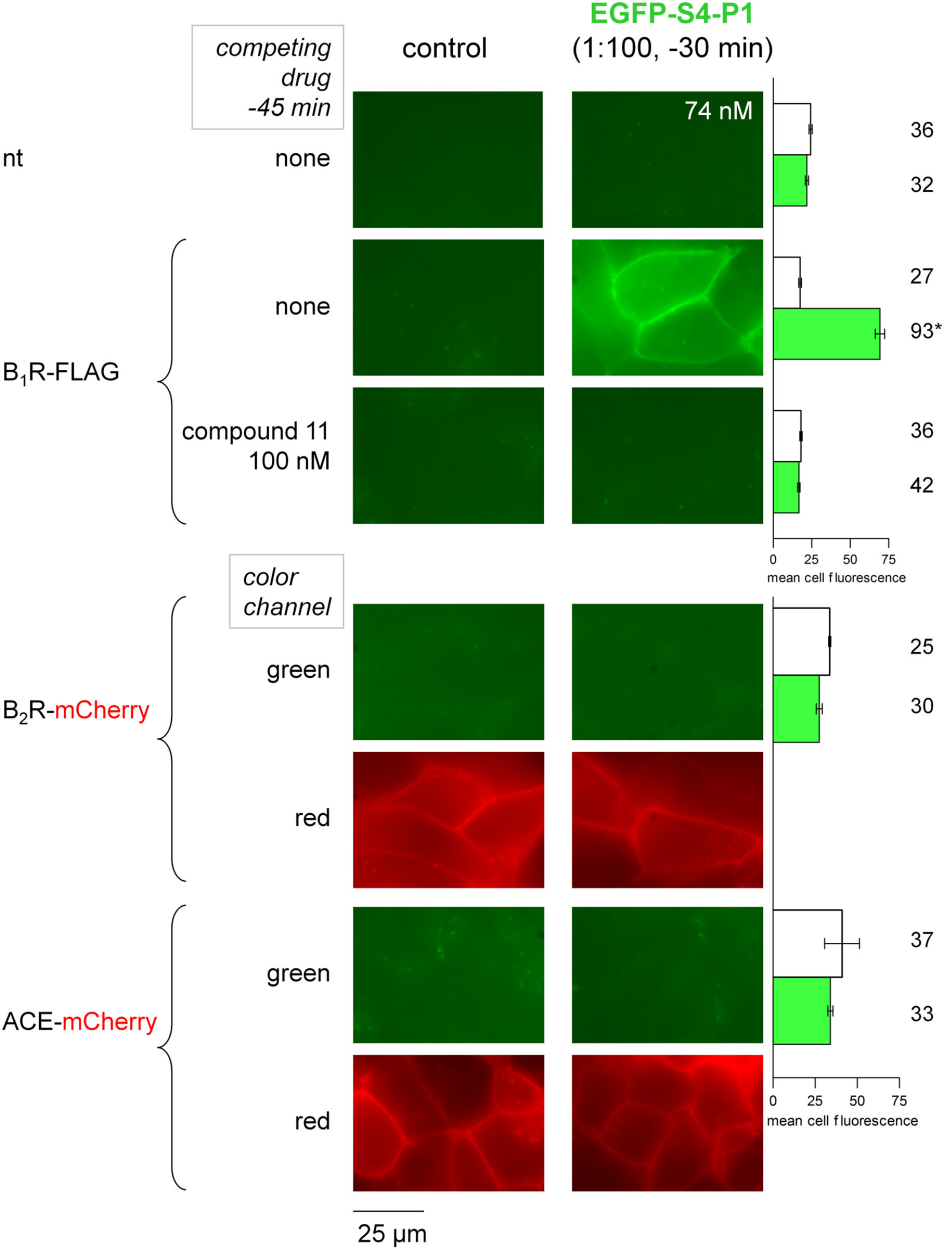
Characterization of EGFP-S4-P1 labeling of molecular targets related to the kallikrein-kinin system expressed in HEK 293a cells. Top: competition of labeling by co-treatment with the non-peptide B_1_R antagonist coumpound 11. In this set of experiments, the 1:200 dilution of the producer cell lysate also produced a good labeling of B_1_R-FLAG, but the staining was weak with the 1:1000 dilution (data not shown). Top right: white histograms represent the averaged cellular fluorescence intensities of cells incubated without ligand, whereas green histograms were from EGFP-S4-P1 cells. ANOVA indicated that the mean cellular fluorescence intensities were heterogeneous (P<10^−4^). * Value different from all 5 others (P<0.001, Tukey-Kramer multiple comparison test), whereas all other pairs of values were not significantly different between them. Bottom: Lack of binding to other molecular targets related to the kallikrein-kinin system. Human B_2_R and ACE were fused to the mCherry FP; each construction was validated by the binding of its cognate radioligand. The red fluorescence allowed the visual confirmation of expression at the plasma membrane. Representative results of multiple microscopic fields. Bottom right: ANOVA indicated that the mean cellular fluorescence intensities were homogeneous (P = 0.48).

The pharmacological profile of the optimal ligand EGFP-S4-P1 was studied in additional experiments in which cells expressing hB_1_R-FLAG or not were pretreated with the non-peptide B_1_R antagonist compound 11 [6]. The latter drug, at 100 nM, prevented the labeling of receptor-expressing cells by the membrane fluorescence associated with EGFP-S4-P1 (Fig 6). This labeling was not observed in non-transfected cells or in cells that expressed recombinant human B_2_R-mCherry or ACE-mCherry fusion proteins (Fig 6). The expression of the two latter constructs at the plasma membrane of transiently transfected cells was verified by their intrinsic red fluorescence (Fig 6) and they were further validated by the high affinity binding of cognate radioligands, [^3^H]BK and [^3^H]enalaprilat, respectively (S1 Fig).

**Fig 7:**
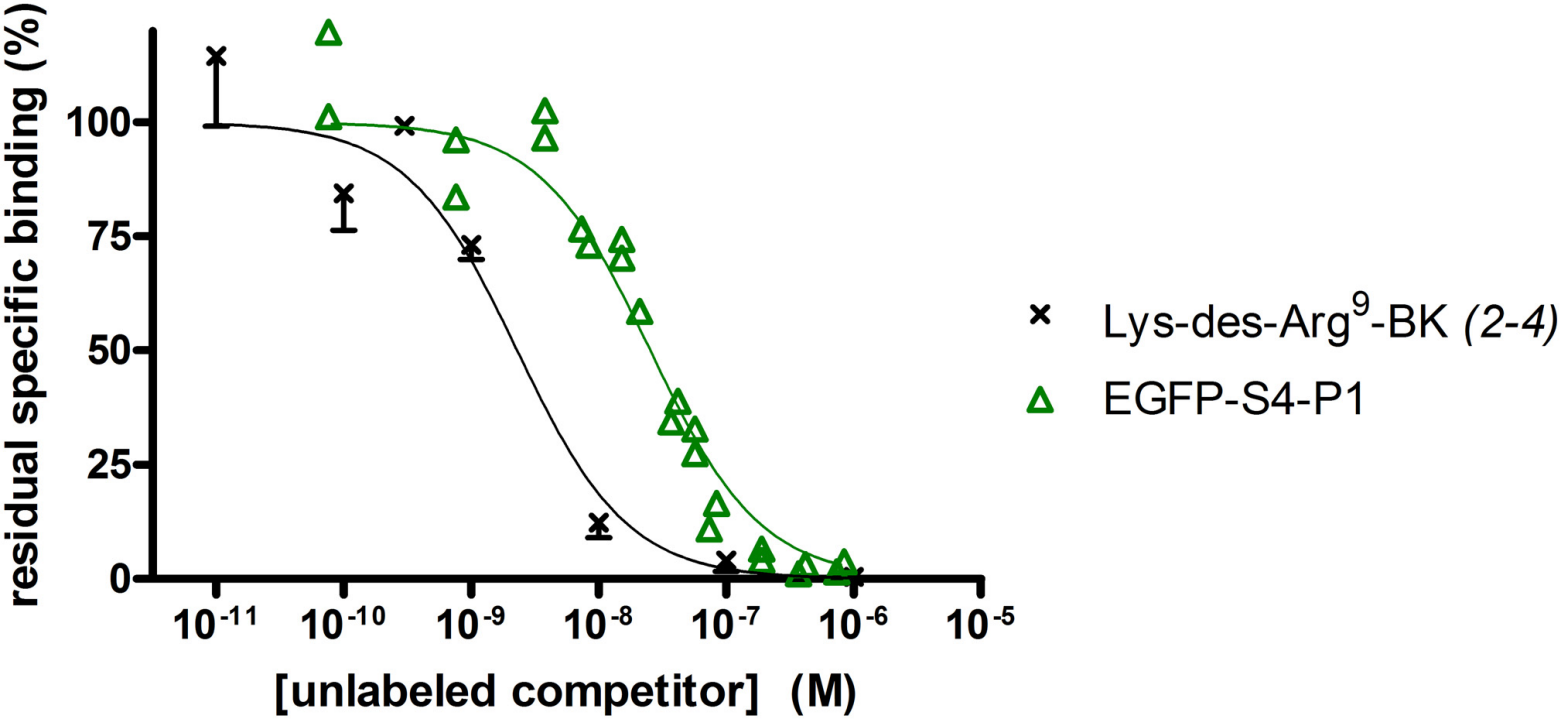
Competition of [^3^H] Lys-des-Arg^9^-BK (1 nM) binding to hB_1_R-FLAG by EGFP-S4-P1, the most potent construction (10-fold less potent than unlabeled Lys-des-Arg^9^-BK). Presentation as in Fig 2. At each concentration, the values of residual binding for the Lys-des-Arg^9^-BK curve have been averaged, and the S.E.M. shown for the number of determination indicated between parentheses. For EGFP-S4-P1, several preparations of the protein were used, with different protein concentration estimated *a posteriori* with the GFP ELISA. Therefore, all individual replicates are shown, but the curve is generated using the same equation and least-square method.

We returned to the radioligand binding competition assay to assess the receptor affinity of EGFP-S4-P1 at the human B_1_R, using various dilutions of a cell lysate of known concentration (determined by ELISA for GFP; Fig 7). The fusion protein (IC_50_ 25.5 nM, 95% C.L. 20.2–32.2 nM) was only 10-fold less potent than unlabeled Lys-des-Arg^9^-BK (IC_50_ 2.3 nM in this series of experiments, 95% C.L. 1.5–3.5 nM) to displace the tritiated form of the latter ligand, indicating a nanomolar affinity for EGFP-S4-P1 (K_i_ = 6.6 nM estimated using by the Cheng-Prusoff correction from the reported K_D_ of 0.35 nM for the radioligand binding to hB_1_R-FLAG [14]).

The same HEK 293a recipient cells expressing the wild type human B_2_R [16] were treated for 30 min with a lysate containing the B_2_R agonist fusion protein, EGFP-MK. As previously reported for cells expressing the rabbit B_2_R [9], the cells exhibited an essentially endosomal staining pattern, with little residual labeling of the plasma membrane (S2 Fig). Non-transfected cells or cells untreated with EGFP-MK showed essentially the background autofluorescence of cells.

### Effect of EGFP-S4-P1 fusion proteins on naturally expressed B_1_Rs in human umbilical artery smooth muscle cells (hUA-SMCs)

**Fig 8:**
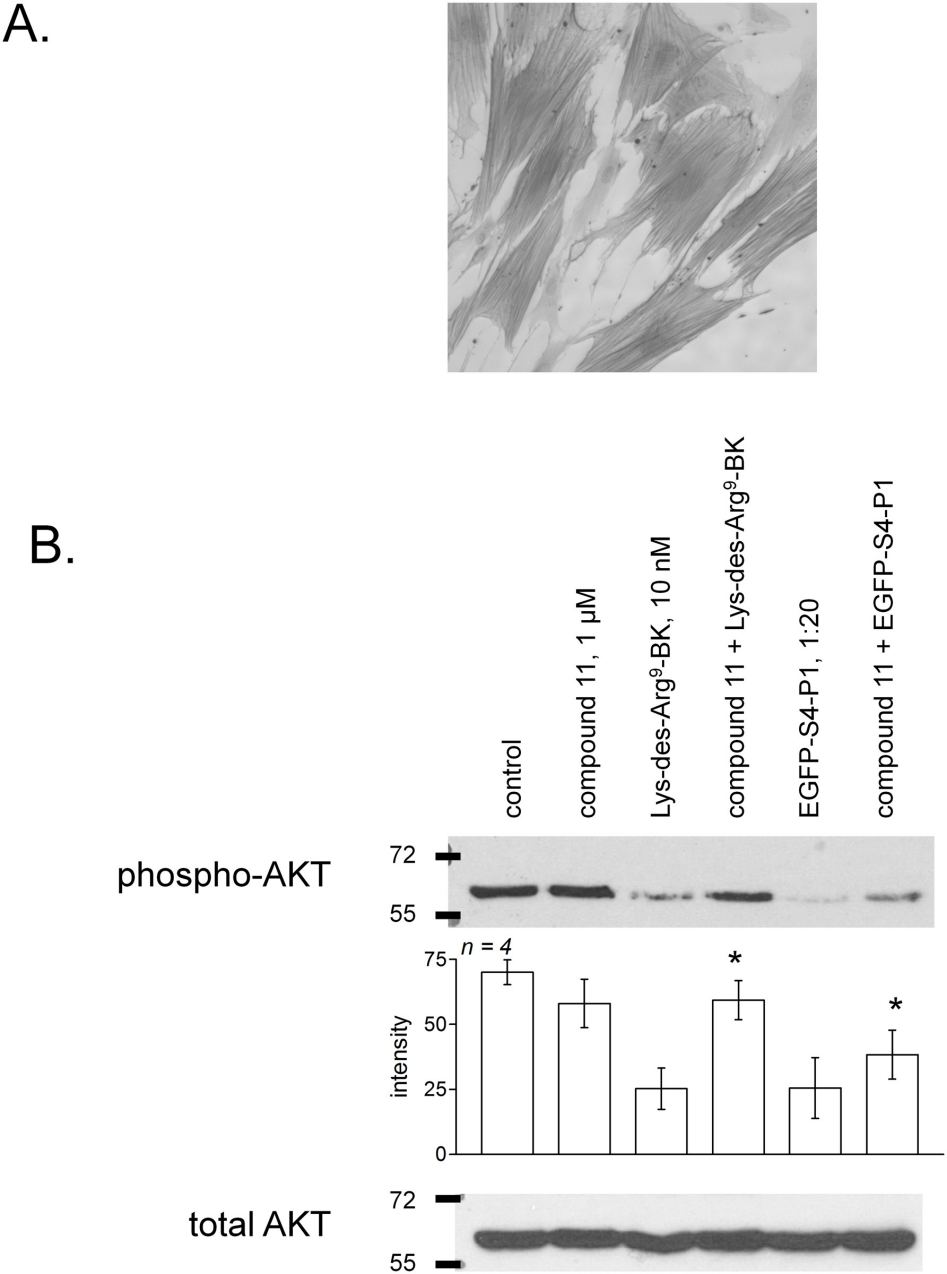
Experiments based on hUA-SMCs. A. Immunohistochemistry of the marker α-actin in permeabilized cells (original magnification 100×). B. Inhibition of AKT phosphorylation following the acute stimulation of endogenous B_1_Rs in hUA-SMCs pretreated for 16 hrs with cytokines (interferon-γ 100 ng/ml + tumor necrosis factor-α 10 ng/ml). The inhibitory effect of either Lys-des-Arg^9^-BK (10 nM) or EGFP-S4-P1 (final concentration estimate 441 nM) was significantly reversed by co-treatment with the B_1_R antagonist, compound 11 (* P<0.05, effect of compound 11 vs. each stimulation condition including control, paired t test).

hUA-SMCs express a regulated and functional endogenous population of B_1_Rs [6, 7, 15]. The acute suppression of AKT phosphorylation is a documented signaling effect of B_1_R agonists in these cells [15], and this has been replicated in cells pretreated with cytokines to upregulate receptor expression (Fig 8). The inhibitory effect of Lys-des-Arg^9^-BK was reversed by co-treatment with the antagonist compound 11; EGFP-S4-P1 inhibition of AKT phosphorylation was also significantly reversed by compound 11. However, EGFP-S4-P1 did not support cell membrane B_1_R imaging (epifluorescence) in cytokine-treated hUA-SMCs (data not shown). Evidence based on [^3^H]Lys-des-Arg^9^-BK binding shows that the latter cells express approximately 2.4 fmol of B_1_Rs per cm^2^ of culture surface [7], whereas recombinant B_1_R-FLAG is expressed at approximately 9.5 fmol/cm^2^ in transfected HEK 293a cells (as in Figs 2 and 7).

## Discussion

Using a rational design strategy, we generated the nanomolar affinity fusion protein EGFP-S4-P1 as a ligand of the bradykinin B_1_R (minor loss of affinity vs. the optimal agonist of the human B_1_R). This design is an agonist; fusion proteins that contain the antagonist ligand (P2) fared poorly due to the intrinsically lower affinity of antagonists (Fig 2), but also possibly to agonist-induced modulation of subcellular distribution because intact cells are treated at 37°C with the fusion proteins before microscopic observation. Indeed, the plasma membrane B_1_R has been proposed to be rapidly internalized and degraded in a ligand-independent manner, but is paradoxically stabilized at the plasma membrane level when bound to the agonist [20]. Alternatively, the signaling-induced translocation of B_1_Rs from the secretory pathway to the cell surface is possible. The des-Arg form of the high affinity B_2_R probe EGFP-maximakinin (EGFP-S1-P1) has little affinity for the B_1_R. EGFP-S4-P1 is a specific probe for the B_1_R as demonstrated by the lack of binding in the presence of compound 11, a specific non-peptide B_1_R antagonist, or in the absence of the receptor. It contains a long spacer, (NG)_15_, previously used in other constructions and known to be compatible with the extracellular fluid. It retains Lys^0^ of the optimal agonist Lys-des-Arg^9^-BK of the human B_1_R. Further, current results support that EGFP-S4-P1 is highly selective for the BK B_1_R as the fusion protein did not bind to cells that expressed the B_2_R or the kinin-destroying peptidase ACE. The latter enzyme is known to metabolize des-Arg^9^-BK [21] and may be ~70-fold more abundant than cytokine-upregulated B_1_Rs in cultured human umbilical vein endothelial cells, based on B_max_ values from radioligand binding assays [7, 14]. EGFP-S4-P1 is probably excluded from the catalytic sites of ACE, being a fairly large protein. Thus, the fusion protein achieves a better fluorophore stability and target specificity than small peptides labelled with chemical fluorophores. Further, spacers exhibit empirically discovered specificities for kinin receptor subtypes: the MK design (S1) is incompatible with B_1_R binding, but EGFP-MK binds to both human and rabbit B_2_Rs (S2 Fig) [9]. Conversely, a FP-S4-BK construction failed to bind to the human B_2_R (data not shown), showing that S4 performs best in B_1_R constructions. Thus, both FP-MK and FP-S4-P1 types of constructions could be used in the same experiments with little risk of confusion between the 2 receptor subtypes.

The BK B_2_R is phosphorylatable by G protein coupled kinases and then associates with β-arrestins [1] whereas the B_1_R has no conserved C-terminal phosphorylation domain. The B_1_R does not translocate cytosolic β-arrestin_2_ when activated, unlike the B_2_R (microscopy) [4]. Using GFP-conjugated ligands, we confirmed that plasma membrane / endosomal staining of B_1_R and B_2_R agonists differ between them in live cells (Figs 4–6, S2 Fig), but are similar to those of chemical fluorophores conjugated to their respective agonist peptides [3, 8]. However, recombinant ACE was labeled by the BK analog, not by EGFP-MK [9].

The bradykinin B_1_R mRNA is upregulated in blood CD4^+^ lymphocytes from patients diagnosed with active multiple sclerosis [22]. Peripheral blood mononuclear cells also express B_1_R mRNA in acute coronary syndrome and cardiac syndrome X [23, 24], vascular disorders where an inflammatory component is increasingly documented. Whether the expression of B_1_Rs has any relevance for the diagnosis of such disorders using cytofluorometry remains to be seen and, if so, FP-S4-P1 ligands may prove useful. Cancer lesions also frequently overexpress the B_1_R and various conjugated synthetic antagonist peptides have been recently used for the in vivo radioimaging of tumors; the kidneys and urinary tracts of animals that have received these tracers are also typically strongly labeled [10, 25]. This suggests a possible advantage of higher molecular proteins that evade glomerular filtration as probes and, perhaps, as carriers of cytotoxic cargoes targeted to the lesions.

Limitations of the current approach include the lack of detection of naturally expressed B_1_Rs in hUA-SMCs by the best fusion protein ligand, EGFP-S4-P1, consistent with the usual lower detection limit of radioactive tracers *vs*. fluorescent ones. Increased detection sensitivity may be reached by using a FP more intensely fluorescent than EGFP or by replacing FPs with an enzyme such as horseradish peroxidase or biotin ligase in modular biotechnological constructions that retain the S4-P1 C-terminal sequence. Further, as discussed above, the agonist properties of the best ligand may acutely alter the subcellular distribution of the B_1_Rs. Finally, affinities of EGFP-S4-P1 for all possible cellular receptors (e.g., scavenger receptors) or non-specific binding sites present in plasma proteins have not been determined.

This study identifies the modular C-terminal sequence S4-P1 that can be adapted to protein cargoes, conferring high affinity for the BK B_1_R, with possible applications in diagnostic cytofluorometry, histology and drug delivery (e.g., in oncology).

## Supporting Information

S1 Fig Validation of mCherry-conjugated molecular targets using radioligand binding assays applied to intact adherent cells. A. Saturation of [^3^H]BK binding in HEK 293a cells that transiently expressed human B_2_R-mCherry fusion protein, or in mock-transfected cells (methods as in [8]). B. Saturation of [^3^H]enalaprilat binding in HEK 293a cells that transiently expressed ACE-mCherry, or in mock-transfected cells (methods as in [14]). Values are the average of specific binding obtained in duplicate. Curves were fitted to the equation B_max_×X/(X+K_D_), where X is the radioligand concentration, using a least-square methods (Prism 4.0, GraphPad Software Inc., San Diego, CA). Parameters for [^3^H] binding to B_2_R-mCherry: K_D_ = 4.1 nM (95% C.L. 1.90–6.24), B_max_ = 37.82 fmol/well (95% C.L. 27.4–48.2); for [^3^H]enalaprilat binding to ACE-mCherry: K_D_ = 3.30 nM (95% C.L. 1.96–4.64), B_max_ = 151 fmol/well (95% C.L. 121–181).
(TIF)

S2 Fig Microscopic labeling of HEK 293a cells that transiently express the wild type human B_2_R using a lysate of other producer cells that expressed EGFP-MK. Cells were treated for 30 min at 37°C with fusion protein in the form of the diluted (1:67) lysate of other producer cells. The staining is essentially endosomal. Presentation as in Fig 4.
(TIF)

## Abbreviations

ACE: angiotensin converting enzyme
B_1_R: B_1_ receptor
B_2_R: B_2_ receptor
BK: bradykinin
dsDNA: double-stranded DNA
EGFP: enhanced green fluorescent protein
FP: fluorescent protein
GFP: green fluorescent protein
hUA-SMC: human umbilical artery smooth muscle cell
mCherry: monomeric cherry fluorescent protein
MK: maximakinin
PCR: polymerase chain reaction
