# Supplementary figures and images for: Biotechnological Fluorescent Ligands of the Bradykinin B1 Receptor: Protein Ligands for a Peptide Receptor
Source: PLoS One. 2016 Feb 4;11(2):e0148246. doi: 10.1371/journal.pone.0148246 (PMC4742067; doi:10.1371/journal.pone.0148246)

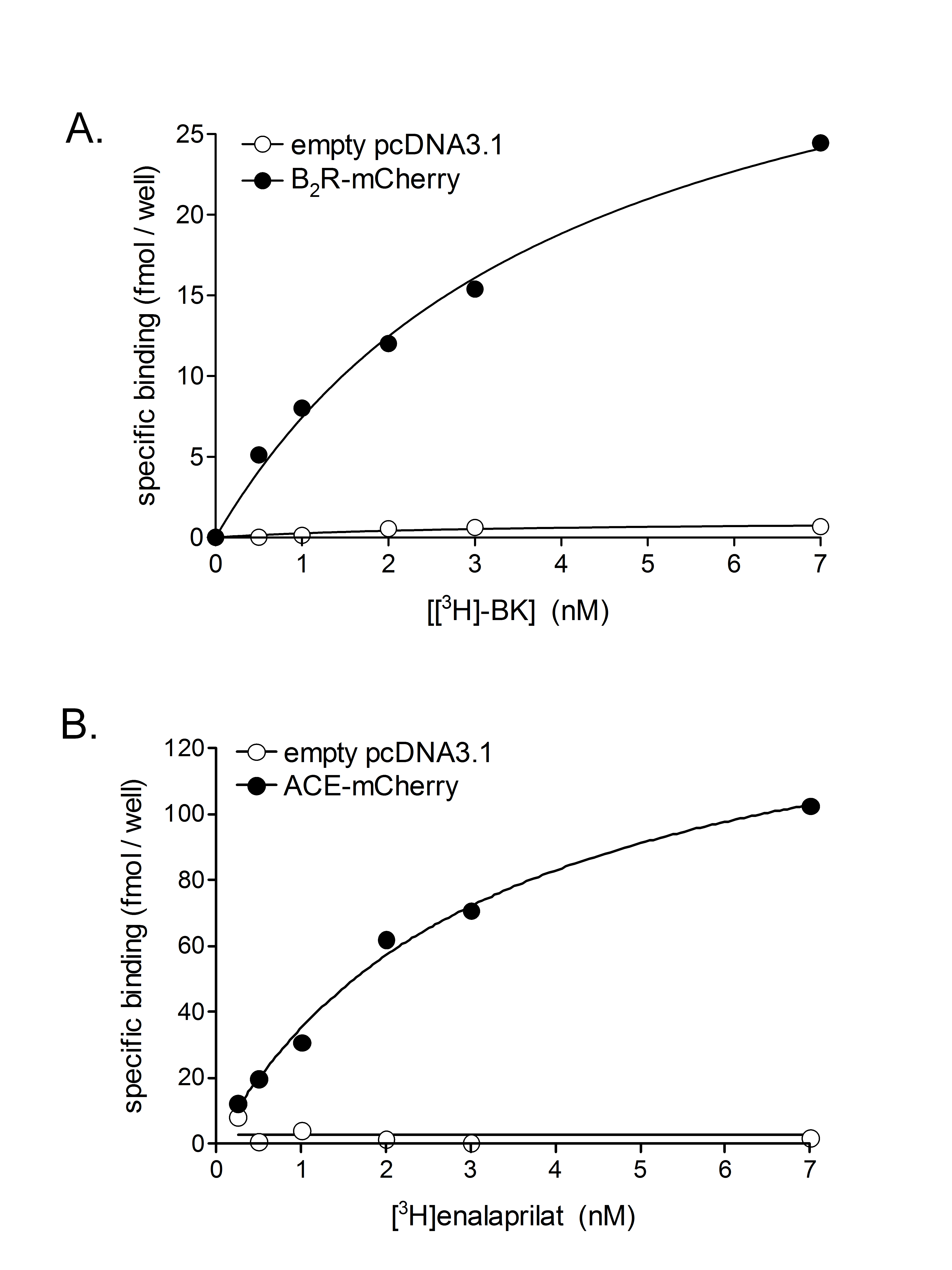

Supplement: S1 Fig — A. Saturation of [3H]BK binding in HEK 293a cells that transiently expressed human B2R-mCherry fusion protein, or in mock-transfected cells (methods as in [8]). B. Saturation of [3H]enalaprilat binding in HEK 293a cells that transiently expressed ACE-mCherry, or in mock-transfected cells (methods as in [14]). Values are the average of specific binding obtained in duplicate. Curves were fitted to the equation Bmax×X/(X+KD), where X is the radioligand concentration, using a least-square methods (Prism 4.0, GraphPad Software Inc., San Diego, CA). Parameters for [3H] binding to B2R-mCherry: KD = 4.1 nM (95% C.L. 1.90–6.24), Bmax = 37.82 fmol/well (95% C.L. 27.4–48.2); for [3H]enalaprilat binding to ACE-mCherry: KD = 3.30 nM (95% C.L. 1.96–4.64), Bmax = 151 fmol/well (95% C.L. 121–181). (TIF) [file pone.0148246.s001.tif]

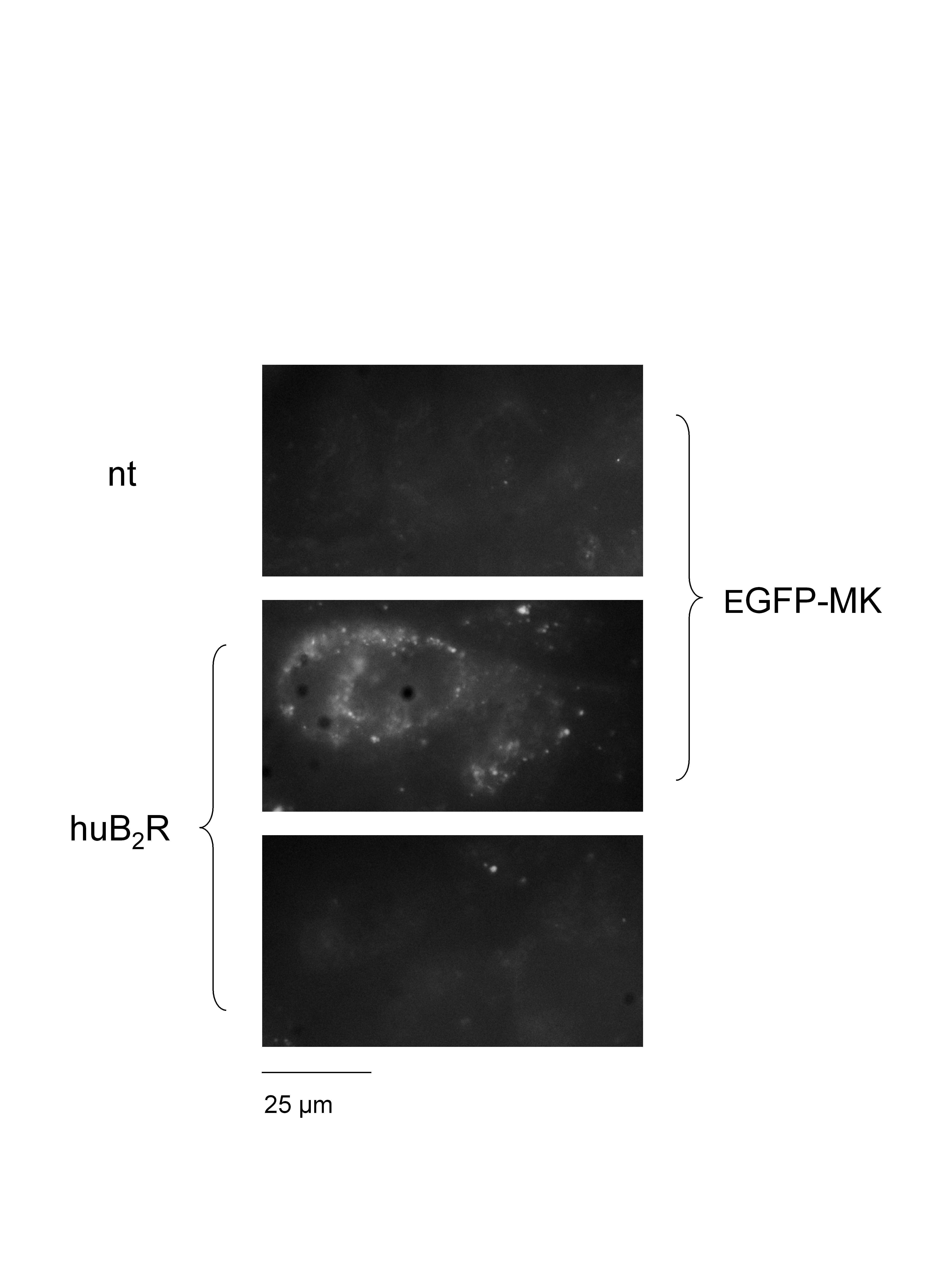

Supplement: S2 Fig — Cells were treated for 30 min at 37°C with fusion protein in the form of the diluted (1:67) lysate of other producer cells. The staining is essentially endosomal. Presentation as in Fig 4. (TIF) [file pone.0148246.s002.tif]
